# Pathology of the Salpinx: A Retrospective Literature Review

**DOI:** 10.7759/cureus.67624

**Published:** 2024-08-23

**Authors:** Itunujesu J Olonade, Adeola Adegunle, Suma Maj Kaza

**Affiliations:** 1 Obstetrics and Gynecology, Avalon University School of Medicine, Willemstad, CUW; 2 Pathology, Avalon University School of Medicine, WIllemstad, CUW; 3 Pathology, Avalon University School of Medicine, Willemstad, CUW

**Keywords:** fallopian tube prolapse, ovarian cancer, ectopic pregnancy, female genital tuberculosis, fallopian tube endometriosis, fallopian tubes

## Abstract

The fallopian tube is a common surgical specimen, yet there is limited research on the histomorphologic findings. This study seeks to review the various abnormalities found in the fallopian tube and establish the primary disease processes linked to it. These findings can provide valuable insights for future preventive healthcare measures.

Utilizing PubMed, a search was conducted for articles published between 2009 and 2024 to investigate fallopian tube pathologies using case reports. The inclusion criteria focused on patients older than 18 years with confirmed or incidental fallopian tube pathology diagnoses. The study considered both common and uncommon presentations of fallopian tube pathologies, with a primary focus on identifying the presenting symptoms related to these conditions, such as primary infertility, severe abdominal pain, tachycardia, hypotension, and breathlessness (the last three could indicate a surgical emergency with ruptured ectopic pregnancy and subsequent hemoperitoneum).

Fifteen studies were included in this review. The findings revealed three cases of genital tuberculosis, two cases of endometriosis, two cases of fallopian tube prolapse, three cases of ovarian cancer, and four cases of ectopic pregnancy. To confirm the presence of these conditions, histopathological examination was performed using specimens obtained through salpingectomy/salpingostomy. This study effectively highlighted the occurrence of rare presentations associated with common fallopian tube pathologies.

By identifying different pathologies present in the fallopian tube, healthcare professionals can expand the range of existing pathologies that may be considered as potential differential diagnoses. This knowledge is essential in directing patient care and has the potential to improve patient outcomes significantly.

## Introduction and background

In the histopathological laboratory, the fallopian tube is frequently encountered as a surgical specimen [1]. Nevertheless, the literature only sporadically provides descriptions of the histologic findings associated with this specimen. Histopathological abnormalities in the fallopian tube play a significant role in the development of infertility and cancer. Conversely, an undetected ectopic tubal pregnancy can pose a grave risk to the life of the mother [2]. The inflammation of the fallopian tubes may stem from physiological factors, as evidenced by negative cultures, or pathological factors, including bacterial infections or other infectious agents [3,4]. Research has indicated a noteworthy correlation between female genital tuberculosis and a substantial incidence of fallopian tube involvement [5]. Tuberculosis of the salpinx is the most common cause of infertility in the world [6].

Early ovarian cancers, particularly low and high-grade serous carcinomas, and the various precursor lesions (most importantly, serous intraepithelial carcinoma, i.e., STIC) can be specifically found in the fallopian tube [7]. Removing the tubes preventatively during hysterectomy or sterilization could prevent future tubal issues like hydrosalpinx [8]. Additionally, this intervention is expected to protect against tumor development.

In this literature review, we will examine existing research and case reports outlining the different types of pathologies found in the fallopian tube. Histopathology specimens were obtained from salpingectomy, salpingo-oophorectomy, or hysterectomy with salpingo-oophorectomy and tubal ligation. The most common tubal pathology seen include ovarian carcinomas, endometriosis, and genital tuberculosis.

This research aims to identify the diverse range of abnormalities observed in the fallopian tube and determine the prevailing disease processes associated with it. These findings can provide valuable insights for future preventive healthcare measures, including risk-reducing surgeries [9].

## Review

Search strategy and study selection

Databases like PubMed and EBSCO were searched from 2009 to 2024. Database searches combined terms from the following five concepts: (1) fallopian tube pathology or histopathology; (2) ectopic pregnancy; (3) ovarian cancer; (4) genital tuberculosis; and (5) endometriosis and prolapse. We also identified additional trials of relevance from the references in the included papers. These were combined as free words and confirmed as MESH terms. Abstracts of all relevant papers identified by the search strategy were selected for full-text retrieval. The authors of this study independently used the eligibility criteria checklist to apply the inclusion criteria identified below to the papers. Analysis of histopathological data was conducted to examine different disease processes.

Eligibility Criteria

Inclusion criteria: To be included in this review, all articles had to meet the following criteria: (1) case reports and clinical studies; (2) publish date between January 1st, 2009 and January 1st, 2024 with at least five articles within the past five years; (3) age limit was women older than 18 years; (4) studies with all possible disease outcomes related to fallopian tube pathology; and (5) any incidental and pre-existing fallopian tube pathology confirmed through pathology or histopathology.

Exclusion criteria: Articles were excluded if (1) the study design was other than case reports or clinical studies; (2) articles were older than 15 years; (3) the population was younger than 18 years; (4) studies with disease outcomes not related to the fallopian tube.

Measurement and Observation: Outcome Measures

The study focused on outcome measures related to different types of fallopian tube pathologies, which included genital tuberculosis, endometriosis, ovarian cancer, and ectopic pregnancy. Each pathology was examined to determine the presenting symptoms associated with it. Infertility was identified as a key presenting symptom of genital tuberculosis, while pelvic pain, dyspareunia, and abdominal pain were recognized as presenting symptoms of endometriosis. Mass effects such as frequent urination, inguinal swelling, and lower abdominal pain were assessed as presenting symptoms of ovarian cancer. Ectopic pregnancy evaluation involves assessing gravida and parity, as well as relevant past medical history like sexually transmitted infections (STIs). The diagnostic method employed was confirmation of disease from specimens obtained through salpingectomy.

Data Extraction

Two reviewers worked independently to extract the following information: study design, study demographics (age, sex, gender, and race/ethnicity if included in the study), eligibility criteria, method of diagnoses, type of pathology identified, and outcome measures. General characteristics about authors, first author, and publication year were also extracted. The results for outcome measures were prevalence. Disagreements on data were resolved by discussion.

Results

Description of Studies

**Figure 1:**
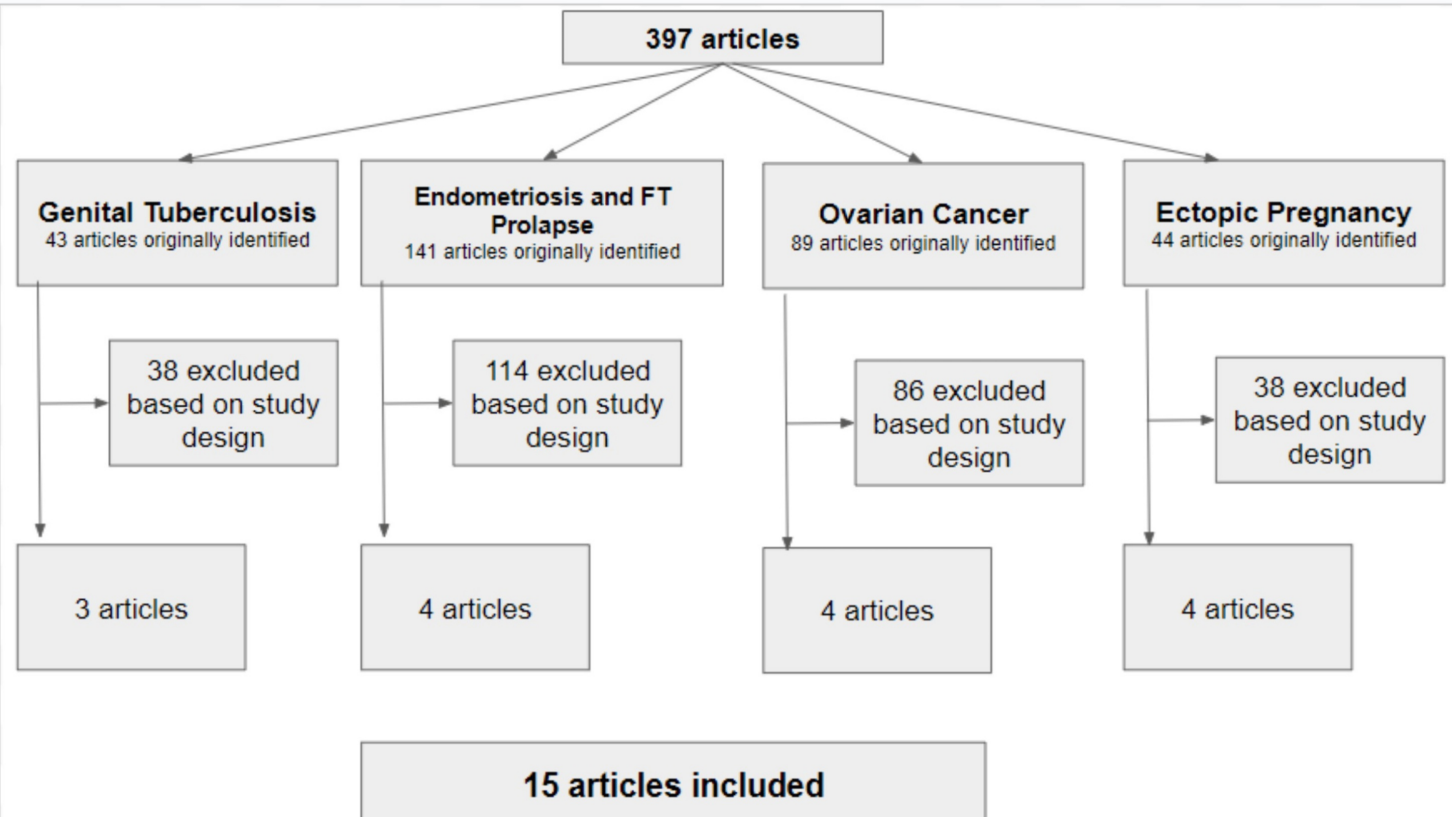
Flowchart of the studies included in the literature review of common fallopian tube pathologies. FT: fallopian tube.

Figure 1 describes how the literature search identified 397 studies based on relevant outcomes and variations of pathologies. The decision was made to combine search terms according to the different pathologies. Detailed abstract screening resulted in 43 articles for fallopian tube pathologies and genital tuberculosis. A total of 38 were excluded based on study design (not being case reports or clinical trials). Main article screening resulted in three articles based on inclusion criteria. Detailed abstract screening for fallopian tube pathologies, endometriosis, and fallopian tube prolapse resulted in 141 articles, and 114 were excluded based on study design and age range. A total of 25 were potentially available for inclusion, and three studies were selected based on inclusion criteria. Abstract screening for fallopian tube pathologies and ovarian cancer resulted in 89 potential articles, with four included based on inclusion criteria. Abstract screening for fallopian tube pathologies and ectopic pregnancy resulted in 44 studies, and 17 were excluded based on study design. In total, 15 articles were included in this study.

Table 1 describes the studies including the type of fallopian tube pathologies, method of obtaining specimens, and demographics.

**Table 1:** Studies included in the literature review of common fallopian tube pathologies.

Author	Fallopian tube pathology	Type of study	Method of obtaining the histopathological specimen	Age	Race/ethnicity
Tzelios et al. [10]	Genital tuberculosis	Case report	Bilateral salpingectomy	33	Asian/Chinese
Wagner et al. [11]	Genital tuberculosis	Case report	Left salpingectomy	23	African/Moroccan
Yang et al. [12]	Genital tuberculosis	Case report	Bilateral salpingotomy	26	Unknown
Aggarwal et al. [13]	Endometriosis	Case report	Left salpingectomy	31	Unknown
Marujo et al. [14]	Endometriosis	Case report	Left salpingectomy	38	Unknown
Goyal et al. [15]	Prolapse	Case report	Excision of supposed mass	24	Asian/Indian
Bedaiwy et al. [16]	Prolapse	Case report	Salpingectomy	35	Unknown
Murakami et al. [17]	Ovarian cancer	Case report	Total hysterectomy and bilateral salpingo-oophorectomy	72	Asian/Japanese
Hong et al. [18]	Ovarian cancer	Case report	Total hysterectomy and bilateral salpingo-oophorectomy	48	Unknown
Amaral et al. [19]	Ovarian cancer	Case report	Total hysterectomy and bilateral salpingo-oophorectomy	34	Unknown
Maeda et al. [20]	Ovarian cancer	Case report	Total hysterectomy and bilateral salpingo-oophorectomy	77	Unknown
Ngene and Lunda [21]	Ectopic pregnancy	Case report	Right salpingectomy	35	Unknown
Sekar et al. [22]	Chronic ectopic pregnancy	Case report	Left salpingectomy	31	Caucasian/British
Shen et al. [23]	Ectopic pregnancy	Case report	Right salpingectomy	22	Unknown
Tankou et al. [24]	Bilateral ectopic pregnancy	Case report	Bilateral salpingectomy	40	African/Cameroonian

In the study, there were three cases of genital tuberculosis, two cases of endometriosis, two cases of fallopian tube prolapse, three cases of ovarian cancer, and four cases of ectopic pregnancy. The age range of the participants varied from 23 to 77 years old. Among the studies, six of them provided information about the race of their participants, which consisted of individuals of African, Asian, and European descent. On the other hand, eight studies did not include any details regarding the race or ethnicity of the patients. Out of the 15 articles, 14 of them collected their specimens through salpingectomy, while one case report opted for salpingotomy to preserve fertility.

Common Fallopian Tube Pathologies

All studies included in this review addressed common fallopian pathology. The first study by Tzelios et al. [10] reported a 33-year-old Chinese woman who presented with primary infertility with a history of tuberculosis. A hysterosalpingogram revealed a rigid right fallopian tube and a nodular left fallopian tube with multiple strictures. Pathology did not reveal acute tuberculosis infection but there was transmural endometriosis and focal obliteration of the fallopian tube. There were also areas of fibrosis and chronic inflammation in the parametrium and pelvic side wall. These findings in addition to her history suggested tuberculosis as the major contributor to her infertility. Sutherland et al. write that there is some existing evidence with limited data to suggest sexual transmission of tuberculosis as a primary infection [25].

Wagner et al. [11] discussed a 23-year-old woman from Morocco who presented with one year of intermittent abdominal pain, weight loss, and dyspareunia. Initial laboratory investigations showed elevated CA-125 of 224.6 (range: <35kU/L) and an increased Risk of Ovarian Malignancy Algorithm (ROMA) index of 13.2%. Transvaginal ultrasound revealed edematous bilateral fallopian tubes and a cystic mass near the right ovary. Exploratory laparoscopy revealed few adhesions between the abdominal wall and pelvis and multiple miliary nodes all over the fallopian tubes. Left salpingectomy was performed and histopathological investigation revealed necrotizing granulomas consistent with tuberculosis.

Yang et al. [12] described a 26-year-old who presented with right lower abdominal pain for eight days. The patient had a history of a 3-cm adnexal mass that was diagnosed six years prior via ultrasound. Rectal examination showed a cystic mass at the right adnexa. Ultrasound confirmed the mass to be 7.2 x 7.0 cm in the right ovary. Upon laparoscopic evaluation, diffuse miliary white nodules were seen on the surface of the peritoneum, liver, omentum, and diaphragm. The ampulla of the bilateral fallopian tubes was hard and segmented. Intraoperatively, the right ovary mass was confirmed to be 10 x 8 x 7 cm, and the fallopian tubes were thickened and contained a gray-white cheesy material. Histopathology confirmed a right ovarian benign serous cystadenofibroma and chronic granulomatous inflammation. Polymerase chain reaction and acid-fast bacilli were positive for tuberculosis.

Aggarwal et al. [13] reported a 31-year-old primigravida who presented to the labor ward at 21 6/7 weeks with severe upper abdominal pain and vomiting. This was a dichorionic-diamniotic twin pregnancy conceived via in vitro fertilization due to primary infertility as a result of endometriosis. Magnetic resonance imaging (MRI) revealed one viable fetus and a second non-viable fetus. The patient was initially managed conservatively, but three days after admission, she developed worsening abdominal pain and shortness of breath. Urgent exploratory laparotomy revealed a hemoperitoneum of 2.2 L and a bleeding-distended left fallopian tube due to endometriosis. Endometrial deposits were also seen on the fundus, posterior uterus, and terminal ileum. Left salpingectomy was performed and histology confirmed hemorrhagic foci of endometriosis.

Marujo et al. [14] reported a 38-year-old nulliparous woman with a history of primary infertility and chronic pelvic pain for three years. Her symptoms included dyspareunia, dyschezia, and occasional rectal bleeding. A bimanual examination revealed a tender rectocervical mass involving the uterosacral ligament. Diagnostic laparoscopy showed a frozen pelvis with an adherent right ovary and a non-visualized left ovary. Upon further lysis of the adhesions, it was discovered that the left fallopian tube had pierced the rectum and the distal fallopian tube was in the endoluminal position. Left salpingectomy was performed and histological diagnosis confirmed tubal endometriosis and chronic salpingitis but no endometrial deposits in the rectal segment.

Goyal et al. [15] reported a 24-year-old Punjabi woman with a fleshy mass protruding from a midline vertical abdominal scar. The patient had undergone a cesarean section six months prior for a term delivery of a breech presentation. The postoperative course was complicated by wound dehiscence on day six, which was treated conservatively. She presented six months later with a 2 x 2 cm fleshy mass protruding from the abdomen. The patient also reported monthly menstrual bleeding from the mass for the past five months. This raised high suspicion for possible endometriosis of the scar. Surgery for excision of the mass revealed the left side of the uterus adhered to the abdominal wall and the fimbrial end of the fallopian tube was protruding through the abdominal scar mimicking the endometriosis deposit. The presence of the fallopian tube was confirmed by passing a probe through the tube.

Bedaiwy et al. [16] reported a 35-year-old G2P2 who presented with symptoms of heavy vaginal bleeding with clots, and a mass protruding from the vagina. Relevant history includes multiple fibroids treated with hysteroscopy and myomectomy. The patient had recently undergone a hysterectomy for a prolapsed fibroid with a complicated postoperative course. One month later, during a routine Pap smear exam, a soft pink tubular structure was seen at the external os. Fallopian tube prolapse was diagnosed. A laparoscopy revealed the left fallopian tube was buried under intra-abdominal adhesions and a left salpingectomy was performed.

Preneoplastic alterations were identified in the fimbrial epithelium of fallopian tubes excised from women with a hereditary predisposition to high-grade serous carcinoma (HGSC). These alterations are now referred to as serous tubal intraepithelial carcinoma (STIC), which has also been observed in the fallopian tubes of women with non-hereditary HGSC [26]. A correlation has been established between STIC and HGSC, attributed to the presence of the TP53 tumor suppressor gene within the tubal epithelium. Additionally, a generic secretory cell outgrowth (SCOUT) has been detected in the fallopian tube, which is linked to changes in PAX2 expression [27].

Murakami et al. [17] reported a 72-year-old patient with a previous breast cancer history presented with frequent urination. MRI revealed a uterine myoma and a right ovarian cyst. The patient underwent a hysterectomy and bilateral salpingo-oophorectomy for the resection of a uterine myoma, revealing a 5 mm tumor in the right fallopian tube. The tumor displayed papillary and alveolar forms, solid areas, and psammoma bodies, accompanied by significant nuclear atypia and mitosis. Immunostaining results indicated positivity for PAX-8, p16, WT-1, and p53, and negativity for napsin A and carcinoembryonic antigen (CEA), consistent with a diagnosis of HGSC. Additionally, metastasis to the para-aortic lymph nodes was identified.

Hong et al. [18] reported a 48-year-old female patient diagnosed with dermatomyositis who was referred to the gynecology oncology department following a Pap smear that indicated the presence of atypical glandular cells characterized by eccentric hyperchromatic nuclei. She subsequently underwent a total hysterectomy, along with a bilateral salpingo-oophorectomy and pelvic lymph node dissection. The pathological examination revealed a diagnosis of high-grade ovarian serous carcinoma, and STIC was identified within the lumen of the fallopian tube. Immunohistochemical staining demonstrated positivity for PAX8 and P53, with the p53 signature also observed in the fallopian tube.

Amaral et al. [19] reported a 34-year-old woman who presented with lower abdominal pain for one year. Physical examination showed a painful left and midline pelvic mass. Laboratory values showed elevated serum levels of CA125 at 122 (reference <35 U/mL) and a ROMA index of 66%. MRI of the abdomen and pelvis showed a heterogenous mass suggesting a primary fallopian tube or ovarian tumor. A total abdominal hysterectomy with bilateral salpingo-oophorectomy and para-aortic node dissection was performed. Histopathology revealed a synchronous endometrioid and ovarian cancer.

Maeda et al. [20] reported a 77-year-old postmenopausal woman who presented with inguinal swelling. Examination revealed a 12 cm mass in the right inguinal region. CA125 and CA19 were in the normal range. Initial histopathology revealed a poorly differentiated adenocarcinoma with hemorrhage and necrosis. Immunohistochemical staining was positive for PAX-8 and WT-1 with overexpression of p53, suggesting that the tumor was of gynecological origin. Further investigation via a total hysterectomy with bilateral salpingo-oophorectomy revealed mild edema of the fallopian tube and peritoneal washings, which were positive for adenocarcinoma. Histopathological analysis of the fallopian tube revealed a primary tumor in the left fimbria and a papillary adenocarcinoma with stromal invasion. The final diagnosis was fallopian HGSC with massive inguinal metastasis.

Ngene and Lunda [21] reported a 35-year-old G4P3 at 16 weeks who presented with sudden severe lower abdominal pain but no vaginal bleeding. Vitals were stable but hemoglobin was 6.3 g/dl. Ultrasound showed an empty uterus with a live fetus in the right adnexa. Emergency laparotomy revealed 2.2 L of hemoperitoneum and a slow leaking right ampullary tubal pregnancy. The contralateral fallopian tube, uterus, and ovaries were normal, and a right salpingectomy was performed. Histology confirmed a tubal pregnancy with the placenta attached to the distal fallopian tube and a macroscopically normal fetus. This status of the fetus was surprising as tubal pregnancies are thought to be due to fetal abnormalities or defects in tubal transportation.

Sekar et al. [22] reported a rare case of a chronic ectopic pregnancy. In the existing literature, it typically presents as repeated episodes of hemorrhaging due to the gradual disintegration of the tubal wall, which forms a complex pelvic mass [28]. Chronic ectopic pregnancies account for approximately 6% of ectopic pregnancies [29].

In this case report, a 31-year-old G1P0 presented with a three-month history of lower abdominal/suprapubic pain. Notably, she had a positive urine pregnancy test three months prior, followed by an episode of heavy vaginal bleeding and pelvic pain and a subsequent negative urine pregnancy test. Due to these events, she presumed she had a miscarriage. It should be noted that the pregnancy was never confirmed on an ultrasound. Further evaluation revealed an elevated white count and C-reactive protein. A transvaginal ultrasound revealed a right hematosalpinx and left hydrosalpinx and a solid vascular structure inferior to the left hydrosalpinx. The patient was treated for a supposed pelvic infection and discharged home with oral antibiotics with a follow-up planned in six weeks. Follow-up ultrasound showed a 3.8 x 3.7 x 3.4 cm mass in the isthmus of the left fallopian tube. The mass appeared to contain thick, cystic, and echogenic trophoblastic tissue and a blood-filled left fallopian tube. Exploratory laparoscopy, which was converted to a laparotomy due to pelvic adhesions, and a left salpingectomy was performed. Histological analysis confirmed the diagnosis of a chronic ectopic pregnancy.

Shen et al. [23] reported a 22-year-old G2P1 at eight weeks gestation who presented with abdominal pain. Pelvic ultrasound revealed a heterogeneous mass in the right adnexa measuring 2.2 x 2.1 cm, which contained a 1.4 x 0.9 x 1.1 cm gestational sac and a 6 mm embryo with active heartbeats; there was no intrauterine gestational sac. Diagnostic laparoscopy revealed a thickened and ruptured right fallopian tube 3 cm in diameter. A right salpingectomy was performed. Gross pathology showed both seemingly normal and edematous villi tissue. Histopathological examination showed numerous large, edematous irregularly shaped chorionic villi; however, there was also trophoblastic proliferation consistent with a hydatidiform mole. Karyotype evaluation confirmed it was a complete hydatidiform mole. The final diagnosis was a rare twin pregnancy consisting of a tubal ectopic pregnancy of a complete hydatidiform mole and a coexisting embryo.

Tankou et al. [24] reported a 40-year-old G10P9 who presented with a five-day history of worsening lower abdominal pain. Past history was significant for multiple STIs. Further investigations revealed a positive urine pregnancy test, hemoglobin of 7.2 g/dl, and abdominal paracentesis revealed hemoperitoneum. Ultrasound was not performed due to limited resources. Emergency laparotomy revealed an intact left tubal pregnancy and a ruptured right fallopian tubal pregnancy with a hemoperitoneum of 1500 mL. A bilateral salpingectomy was performed.

Discussion

Summary of Main Findings

**Figure 2:**
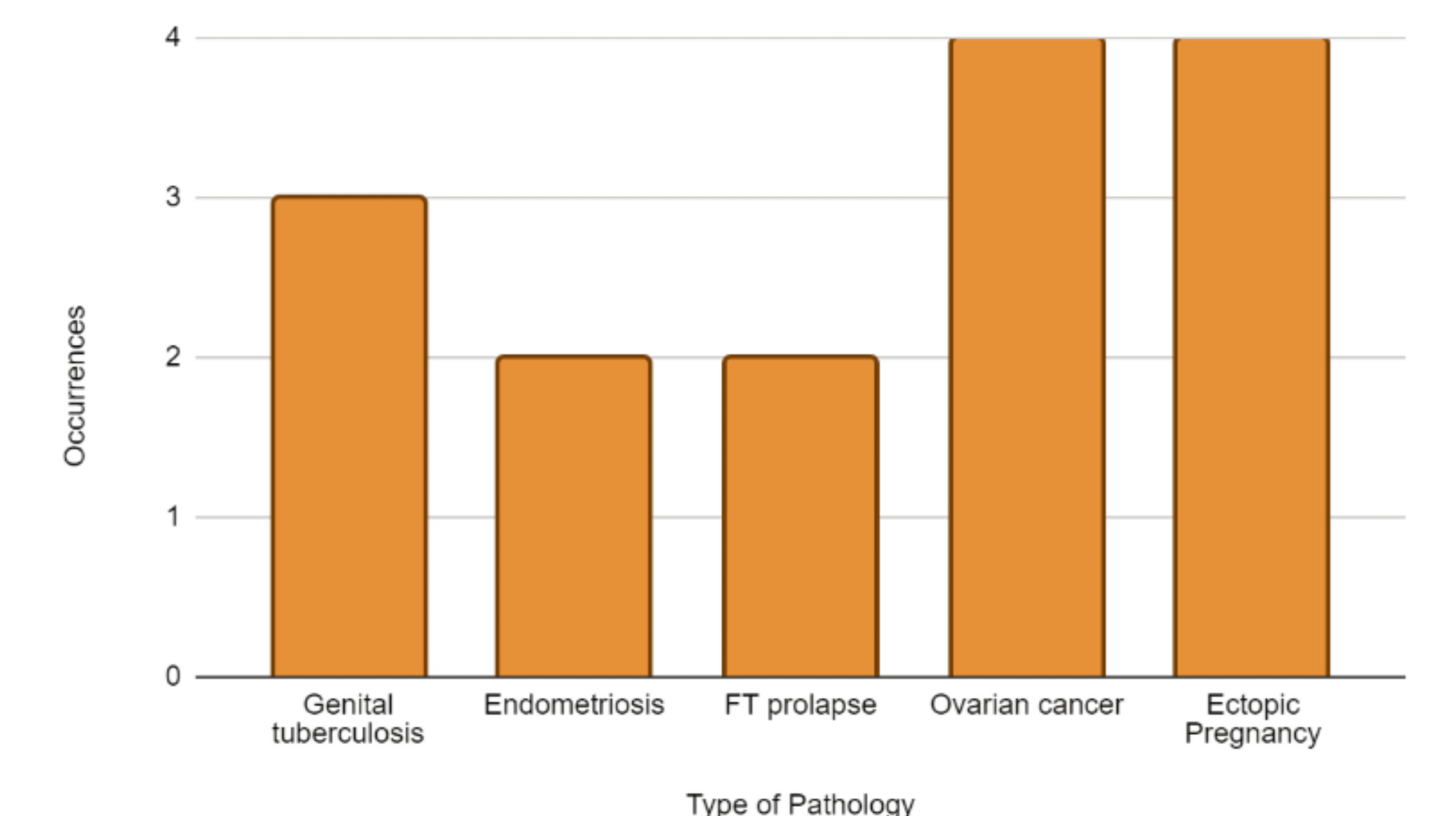
Tabular summary of the studies included in the review. FT: fallopian tube.

According to this review, various fallopian tube pathologies, including ovarian cancer, endometriosis, ectopic pregnancies, and genital tuberculosis, are commonly observed (Figure 2). Familiarity with these conditions can greatly assist in providing optimal patient care, as some of these cases involve uncommon manifestations of well-known pathologies.

When evaluating patients with primary infertility who exhibit the appropriate risk factors, it is crucial to consider genital tuberculosis as a potential differential diagnosis [10,11,25]. The impact of endometriosis on women's health extends beyond their fertility, affecting their daily lives as well. Assessing the spread of endometriosis to the fallopian tubes and understanding its implications can guide the development of effective treatment plans [13,14]. Fallopian tube prolapse, although uncommon, necessitates healthcare providers to be well-informed about its occurrence [15,16]. Additionally, it is important to recognize that fallopian tubes can serve as the site of origin for ovarian cancer, making risk-reducing oophorectomies a viable consideration for patients [17-20]. Ectopic pregnancies, which can be life-threatening and may present atypically, necessitate timely identification and management [21-24].

Strengths and Limitations of This Review

An advantage of this review lies in the reproducibility of the study, as there is a substantial body of literature on fallopian tube pathologies. Further investigations are warranted to enhance the identification of fallopian tube pathologies, with the ultimate goal of informing treatment strategies and improving patient outcomes. Moreover, the study highlights another strength, which is the uniqueness of certain case reports. These reports delve into rare occurrences, inspiring healthcare providers to adopt unconventional thinking when faced with distinctive patient presentations. Consequently, they can consider these cases as potential differential diagnoses.

A drawback of this review is its reliance solely on case reports, leading to a limitation in the variety of information that could be gathered. To enhance the comprehensiveness of the review, incorporating other types of research articles like clinical trials, clinical studies, and retrospective cohort studies would have been beneficial. A further limitation arises from the difficulty in selecting specific articles to be incorporated into the study, given the abundance of studies that meet the inclusion criteria. Consequently, this may result in an arduous and time-consuming process of reviewing numerous articles.

Interpretations of Results

Female genital tuberculosis, including fallopian tube & endometrium, has been identified as an essential causative factor for infertility in places with a significant incidence of tuberculosis, and tuberculosis is seen worldwide. Mycobacterium tuberculosis typically spreads to the genital tract from another primary site in the body, leading to the involvement of bilateral fallopian tubes and/or endometrium [9]. Many individuals with female genital tuberculosis experience mild symptoms and are eventually diagnosed during infertility investigations. This should be taken into account as a potential differential when evaluating primary infertility, particularly in patients from high tuberculosis incidence areas with a risk of exposure. Female genital tuberculosis can also cause elevated CA125 levels, which raises suspicion of ovarian cancer [10]. It should be noted that indirect testing such as tuberculin testing and acid-fast staining may not be sufficient for diagnostics and invasive testing such as diagnostic laparoscopy might be necessary for diagnosis [11,25].

Endometriosis is characterized by the existence of endometrial glands and stroma beyond the confines of the uterine cavity. This condition impacts around 10% of women and is associated with prevalent symptoms such as persistent pelvic pain, infertility, impaired organ function, and psychological distress [10]. One of the case reports emphasizes the considerable influence of endometriosis of the fallopian tube on a twin pregnancy that was achieved through in vitro fertilization. The delayed diagnosis of significant hemoperitoneum could have resulted in an unfavorable outcome for the pregnancy [12]. This delay in diagnosis was attributed, at least in part, to the challenges associated with preoperative diagnosis due to non-specific symptoms and limited available data on the impact of endometriosis on the fallopian tube. These factors should be taken into consideration when managing patients with endometriosis.

A study carried out by De Clippel et al. [30] suggests that fallopian tube prolapse, though infrequent, can develop after a hysterectomy as is also seen in one of the studies in this review [15]. The individual examined in this research was diagnosed with symptoms presumed to be linked to endometriosis [14]. Hence, it is advisable to entertain the prospect of fallopian tube prolapse when faced with similar clinical presentations.

The foremost cause of gynecologic cancer fatality worldwide is ovarian cancer, emphasizing the critical role of prevention and early detection in its therapeutic approach, particularly in instances of HGSC [31]. Growing evidence supports the fallopian tube as a major source of ovarian cancers, thereby shaping patient care protocols. Evidence supports the effectiveness of risk-reducing salpingo-oophorectomy (RRSO) in preventing the occurrence of ovarian cancer among patients with hereditary breast and ovarian cancer syndrome [32]. The fallopian tube can be examined using the sectioning and extensively examining the fimbria protocol.

This recommendation is relevant for women who are undergoing hysterectomies for different clinical indications, as the incorporation of this surgery has been observed to be beneficial for the overall well-being of the patient in reducing the risk of ovarian cancer.

Approximately, 1 to 2% of pregnancies are ectopic, with 90% of these cases happening in the fallopian tube. The majority of these ectopic pregnancies (around 80%) are located in the ampulla of the tube and typically manifest around the 7th week of gestation [33]. This review highlights highly unique occurrences of ectopic pregnancies, specifically twin tubal molar pregnancies [23] and tubal pregnancies surviving past the first trimester [21]. These occurrences are not commonly encountered, and some of the patient presentations exhibited atypical characteristics. The diagnosis of the chronic ectopic pregnancy case proved to be exceptionally challenging, as it was unexpected following what was initially believed to be a miscarriage [22]. Rarely encountered, simultaneous bilateral tubal pregnancies were observed in the patient, who did not manifest the usual symptoms associated with ectopic pregnancy. It was surprising that she maintained hemodynamic stability despite the presence of significant intra-abdominal hemorrhage. The diagnosis and treatment of such cases can be particularly challenging, especially in low-income settings [24].

## Conclusions

Gynecological care holds significant importance within the field of medicine. By identifying different pathologies present in the fallopian tube, healthcare professionals can expand the range of existing pathologies that may be considered as potential differential diagnoses.

The differential diagnoses may include female genital tuberculosis, endometriosis involving the fallopian tube, and the prolapse of the fallopian tube. Moreover, it is essential to consider both high-grade and low-grade serous carcinoma of the ovary, along with the possibility of ectopic pregnancy in the clinical assessment. By maintaining awareness of these differential diagnoses, medical professionals can enhance their diagnostic accuracy and improve patient outcomes. This knowledge is crucial in ensuring that appropriate interventions are undertaken based on the specific pathology identified in the specimens. Most of the pathologies examined in this study were confirmed through histopathological evaluation of specimens obtained via salpingectomy. The significance of this diagnostic technique cannot be emphasized enough, as it provides conclusive answers to a wide range of questions regarding fallopian tube pathologies.
